# Decoding the Hot-Mitochondrion Paradox

**Published:** 2025-07-04

**Authors:** Peyman Fahimi, Michael Lynch, Cherif F. Matta

## Abstract

In a 2018 paper and a subsequent article published in 2023, researchers reported that mitochondria maintain temperatures 10-15 degrees higher than the surrounding cytoplasm - a finding that deviates by 5 to 6 orders of magnitude from theoretical predictions based on Fourier s law of heat conduction. In 2022, we proposed a solution to this apparent paradox. In the present perspective, we build upon that framework and introduce new ideas to further unravel how a biological membrane - whether of an organelle or a whole cell - can become significantly warmer than its environment. We propose that proteins embedded in the inner mitochondrial membrane (IMM) can be modeled as ratchet engines, introducing a novel, previously overlooked mode of heat transfer. This mechanism, coupled with localized heat release during the cyclical dehydration-translocation-hydration of ions through membrane proteins, may generate transient but substantial temperature spikes. In the case of protons, the cycle additionally includes deprotonation before translocation and protonation after. The cumulative effect of these microscopic events across the three-dimensional surface of the IMM can account for the elevated temperatures detected by molecular probes. We also offer a hypothesis based on quantum chemical calculations on how such probes might detect these fleeting thermal signatures.

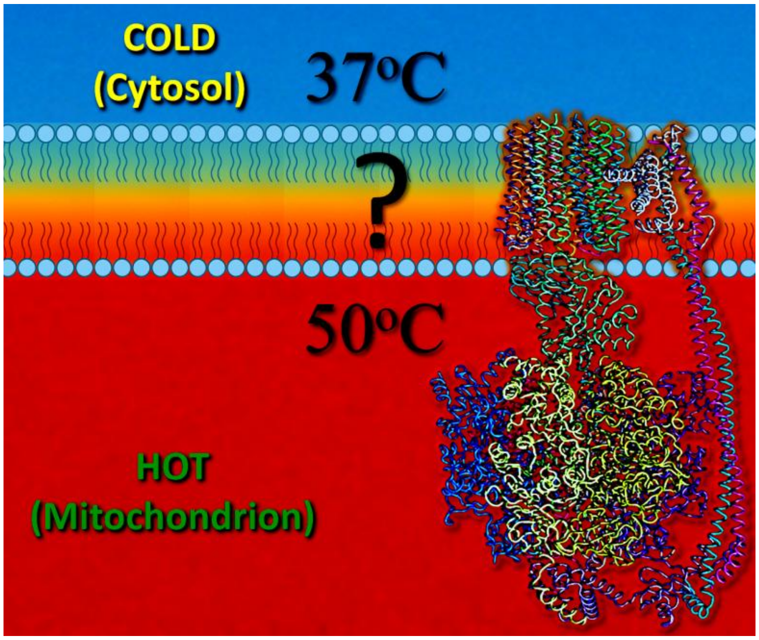

*TOC Graphic 1: All proteins embedded within the inner mitochondrial membrane (IMM) - with ATP synthase shown here as an example - are proposed to function as ratchet engines. Concurrently, the cyclical dehydration-translocation-hydration processes of ions moving between the intermembrane space (IMS) and the mitochondrial matrix act as distributed heat-release events across countless segments of the three-dimensional IMM, collectively shaping the temperature gradient between the mitochondrion and the surrounding cytoplasm.*

## 1. The "hot mitochondrion paradox" summarized in plain language

In 2018, an intriguing article appeared claiming that mitochondria operate at a much higher temperatures than the surrounding cytoplasm, and that mitochondria are, in fact, "hot". These claims are based on interpreting fluorescence intensity measurements of a dye that is known to concentrate inside mitochondria. The paper in question is that of Chrétien *et al.* [1] which was followed-up in 2023 with another paper [2] by the same group confirming their results that mitochondria operate at a temperature ~10ºC – 15ºC higher than their surrounding temperature and extended their results to other experimental model systems. In the very issue of *PLOS Biology* where the 2018 paper appears, a guest-editorial by Nick Lane expressed skepticism regarding claims of a "hot mitochondrion," while not ruling it out entirely [3]. These claims have sparked a heated debate in the literature where, on one side, Chrétien *et al.*'s results were never falsified and still stand unchallenged while steady-state models of heat transfer place a maximal temperature differential at $10^{-5}$ K across dimensions typical of living cells [4–6]. This has been termed the $10^5$ gap in the literature and has remained a paradox until a tentative resolution appeared in 2022 [7]. One of the goals of this article is to simplify this proposed resolution in plain language and to elaborate and extend these ideas. The discovery of temperature gradients within living cells has significant implications to human health and disease. For instance, thermoresponsive nanocarriers can be utilized to deliver anticancer drugs specifically to mitochondria in cancer cells [8]. Furthermore, mitochondrial temperature has the potential to serve as a biomarker for hepatocellular carcinoma [9].

First, we review the literature with particular attention to experimental methods. We then delve into the theoretical framework, building from first principles without technical details (which are available elsewhere [7,10]). Among the early experiments, Okabe *et al.* [11] reported that the temperature of the nucleus and centrosome in COS7 cells is approximately one degree Celsius higher than that of the surrounding cytoplasm. In 2018, Chrétien *et al.* [1,2] proposed that mitochondria operate at significantly higher temperatures than traditionally assumed, based on experiments using a molecular thermometer dye called mito thermo yellow (MTY) [12]. This fluorescent probe predominantly localizes in the mitochondrial inner membrane and its matrix-facing side [1,13]. Contrary to the long-held belief that mitochondrial temperature aligns with the ambient body temperature of 37°C, Chrétien *et al.* suggested that mitochondrial temperatures in human cells could reach up to 50°C. Given that mitochondria are particulate, strategically positioned, and serve as the central hub for ATP production and heat generation (via proton leak [14–17]), it is unsurprising that they are hotter than the average body temperature. However, the real debate is how much hotter.

The extraordinary finding of Chrétien, Rustin, and co-workers has sparked vigorous debate and opened up new avenues for research [3,18]. The problem is that a temperature difference ($\Delta T$) of the order of 10°C over distances of a few nanometers implies astronomically elevated temperature gradients that cannot be maintained with any conceivable material. Nevertheless, the results of Chrétien *et al.* [1,2,13] are reproducible and these workers appear to have taken every possible precaution to mitigate interferences such as the effect of pH or dye concentration on the fluorescence quenching. However, Arai *et al.* [12] emphasized that while factors like pH, viscosity, metal ions, and oxygen species have been tested on MTY in cuvette experiments, these tests do not fully represent the complex molecular environment MTY experiences inside cells, which differs significantly from simple aqueous buffer conditions.

Chrétien *et al.* [1,2,19] obtained their results in human embryonic kidney 293 cells and primary skin fibroblasts with the electron transport chain fully active under various physiological conditions. The conclusions were drawn from observed MTY quenching intensity as a function of temperature, validated through in vitro calibration curves. These researchers invested significant effort in ruling out potential artifacts or interference that might produce false positives. For instance, they demonstrated that the fluorescence decrease was unaffected by either dye concentration or local pH - two critical variables since mitochondrial dye concentrations are difficult to precisely measure and pH across the inner membrane can vary by up to one unit. According to the calibration curve, as also reported in other studies [12], the MTY fluorescence intensity in aqueous solutions decreases by 2.7% per degree Celsius of temperature elevation, independent of pH, oxygen concentration, or ionic strength.

Other researchers have developed distinct methods to map intracellular temperatures across mitochondria, nuclei, lysosomes, and other compartments, obtaining results consistent with those of Chretien *et al*. in mammalian cell lines and yeast cells [20], murine bladder cancer MB49 cells [21], breast cancer MDA-MB468 cells [22], patient-derived tumor samples [23], HeLa cells [24], mouse brains [25], mammalian and insect cell lines

[2], and calcium-induced neurons [26]. Some authors, however, have raised concerns about the reliability of these experiments. For example, real-time temperature mapping of fixed A549 cells (human alveolar basal epithelial adenocarcinoma cells) indicates that localized mitochondrial heating results in a temperature difference of less than 1°C within the cell [27]. Similarly, temperature variations of less than 1°C in the nucleus and cytoplasm of live HeLa cells have been reported [28]. Other experiments have revealed that respiratory complex I becomes unstable at temperatures exceeding 43°C [29]. Concerns have also been raised regarding potential biases in measurements obtained with green fluorescent protein (GFP) nano-thermometers and semiconductor nanocrystals, which require careful consideration [30]. A very recent study using nanodiamond nanothermometry reported that metabolic stimulation does not cause any temperature change in macrophages [31]. The authors also noted that changes in the electrical field on the surface of the nanodiamond could be misinterpreted as temperature variations [31].

A temperature difference of 10–15°C across just a few nanometers of the inner mitochondrial membrane may increase the production of reactive oxygen species (ROS) [32] and proton leak [32], alter membrane structure (including the loss of cristae folds [33] and changes in overall spontaneous curvature [34]), affect lipid phase transitions and fluidity (which are highly dependent on the presence or absence of cardiolipins [35,36]), and destabilize associated proteins [37] - all contributing to mitochondrial dysfunction. How does the mitochondrion modulate or buffer these effects? A thermal stability atlas of 43,000 proteins spanning 13 species, from archaea to humans, reveals that protein melting temperatures range from 30°C to 90°C [37]. Respiratory chain proteins are predominantly stable across species, with human mitochondrial proteins typically functioning at 46°C [37]. A bioinformatics study suggests that the high expression of heat shock proteins (Hsps) in mitochondria plays a crucial role in safeguarding critical macromolecules from melting and mitigating the temperature-induced increase in ROS production [38]. If true, such a significantly higher temperature at which mitochondria operate would require some revision of textbook kinetics and thermodynamics of biochemical reactions happening therein [18].

Going back to the report of Chrétien *et al.* [1,2], a 10°C – 15°C temperature difference across the thickness of the mitochondrial membrane would imply an extraordinarily steep temperature gradient. Unsurprisingly, their claim has been met with skepticism. That skepticism is based on steady-state heat transfer considerations that appear inconsistent by a staggering 5 to 6 orders of magnitude [3–6]. The discrepancy is so substantial that it has acquired the designation of the "$10^5$ gap" between experimental claims and theoretical predictions [3,4].

Fourier's law of heat conduction describes how heat flows from warmer to cooler regions. In its scalar steady-state form, the rate of heat production $\dot{Q}$ (in units of energy per unit time, i.e., watts or joules per second) depends on three factors: the temperature difference $\Delta T$ between two regions, the material's thermal conductivity $\kappa$, which quantifies how easily heat flows through it, and the characteristic length $L$ over which the heat is conducted. In a study by Baffou *et al.* [4], steady-state estimates based on Fourier's law ($\dot{Q} = -\kappa L \Delta T$) suggest that a mammalian cell, modeled as a spherical heat source with a linear dimension of 10 μm and assuming uniform heat generation throughout its volume (i.e., without localized sources such as mitochondria), producing 100 pW of thermal power in a watery environment with $\kappa = 1\ W{\cdot}m^{-1}{\cdot}K^{-1}$, would result in a maximum temperature increase of approximately $10^{-5}$ K relative to its surroundings [4]. Macherel and colleagues [6] corroborated these findings by examining heat transfer mechanisms - conduction, convection, and radiation - and modeling the temperature distribution within an idealized spherical mitochondrion.

How can the rate of heat production per cell be estimated? In heterotrophic eukaryotic cells, the primary source of heat production is mitochondrial proton leak - both basal and inducible [14,15,17,39]. Proton leak refers to the movement of protons from the intermembrane space (IMS) back into the mitochondrial matrix via water wires (WWs), adenine nucleotide translocases (ANTs), and uncoupling proteins (UCPs), bypassing ATP synthase. This process dissipates the Gibbs free energy ($\Delta G$) - the usable energy available to do cellular work - of proton translocation across the IMM as heat. Gibbs free energy, given by the equation $\Delta G = \Delta H - T\Delta S$, reflects the balance between energy released (enthalpy, $\Delta H$) and energy lost to a state of disorder (entropy, $\Delta$S). In mitochondria, this energy is normally used to drive ATP synthesis by moving protons against a pH gradient and electric potential. In this context, the entropy term ($T\Delta S$), where $T$ is the absolute temperature and $S$ is entropy, is negligible compared to the enthalpy term ($\Delta H$) [40]. Therefore, during proton leak, Gibbs free energy and heat can be used interchangeably. Basal proton leak alone accounts for at least 20% of a cell's standard metabolic rate (MR) [14,39]. Therefore, to estimate the rate of heat production per cell, one needs the total metabolic rate - approximately 90% of which is due to mitochondrial oxidative phosphorylation in aerobic cells - and multiply that by the fraction lost as heat through proton leak (i.e., MR × 90% × 20%).

Baffou *et al.* [4] considered the entire cell as a heat source and estimated a temperature increase of only $10^{-5}$ K, revealing a discrepancy of at least $10^5$-fold between theory and experimental observations. This issue is further accentuated by the significantly smaller size of mitochondria, which range from 0.5 to 1 μm, and the metabolic rate per mitochondrion [41,42], estimated to be approximately $3{\times}10^{-4}$ pW in actively growing *Tintinnopsis vasculum* (a ciliate) and up to 1.5 pW in actively growing *Saccamoeba limax* (an amoeba), based on their cellular metabolic rates [43] and mitochondrial populations [44].

In the introduction of his book titled "*Paradoxes*", Sainsbury defines a paradox as: "*an apparently unacceptable conclusion derived by apparently acceptable reasoning from apparently acceptable premises*." [45] (See also Refs: [46,47]). Given the 1-million-fold disagreement between experiment and steady-state theory predictions, we have a paradox whereby the expectation of theory is in total disagreement with experimental observations. What has gone wrong? Is the interpretation of the experimental measurements at fault? Is the theoretical modeling applied outside of its limits of validity?

The present authors termed this situation "*the hot-mitochondrion paradox*" (HMP) and proposed a possible resolution [7]. In the original paper, the proposed resolution of the paradox is rather technical, so we here provide a qualitative and intuitive explanation of its principal points. Building upon our previous theory, we take it a step further, offering a perspective on the potential origins of hot membranes in organelle or cell membranes.

## 2. A resolution of the "hot-mitochondrion paradox" (HMP)

Fourier's law of heat transfer rests on two main pillars: (1) the rate of heat production, and (2) thermal conductance.

1. As briefly discussed in the introduction, the primary source of heat in heterotrophic eukaryotic cells arises from mitochondrial proton leak. This is a long-lived, steady source of heat that dominates the average thermal output of the cell over extended timescales. However, transient heat bursts - much larger in magnitude - occur during ion translocation events. These are typically not counted in the bulk heat production because they average out over time due to the interplay of endergonic reactions in the IMS and exergonic reactions in the mitochondrial matrix (and vice versa), as demonstrated in our previous work [7].

2. The inner mitochondrial membrane (IMM) is highly selective, permitting only specific ions to traverse through designated proteins or enzymes. This selectivity reflects the membrane's resistance to ion flow and thus limits free diffusion of ions between the matrix and IMS. While macroscopic heat transfer is commonly understood to occur via conduction, convection, radiation, and advection, the mechanisms by which heat is transferred through proteins during ion translocation remain poorly understood. Numerous types of proteins have been modeled as ratchet engines in the literature [48–56] - a concept we will elaborate on below. In our previous study [7], we demonstrated that modeling ATP synthase as a ratchet engine results in exceptionally low thermal conductance, implying that ATP synthase conducts heat very weakly. In the present work, we generalize this framework to all IMM-embedded proteins and address the following key questions:

a) What is a ratchet engine, and which proteins in the IMM can be modeled as such?
   b) How are local heat differentials generated by different ions during translocation through the IMM?
   c) How can one illuminate the timescale and length scale of the temperature responsiveness of molecular probes?

A ratchet engine, by definition, consists of a vane and a ratchet enclosed in two separate, thermally isolated boxes filled with gas at two distinct temperatures, $T_1$ and $T_2$. These boxes are mechanically connected via a thermally insulated axle [49,57]. Thus, a ratchet engine involves at least two forms of asymmetry and imbalance: one thermal (i.e., a temperature difference) and the other mechanical or geometric. For simplicity, following the approach developed by Parrondo and Español [58], we consider a model with two vanes instead of a vane and a ratchet. We refer to this simplified setup as the axle-vane engine, as illustrated in Figure 1. This engine operates unidirectionally under non-equilibrium conditions because the temperature - and thus the average kinetic energy of the gas molecules - is higher in one box than in the other (Figure 1). As a result, the Brownian motion of gas particles is more vigorous in the hotter box, leading to an imbalance in random collisions with the vanes. This asymmetry generates a net force that induces unidirectional motion via the rotation of the first vane. This rotation is mechanically transferred to the second vane in the other box via the axle. Consequently, the kinetic energy of the gas molecules in the second box increases, indicating that heat has been transferred mechanically despite thermal isolation. In other words, *heat transfer is not mediated by molecular collisions and vibrations but rather occurs through the classical mechanical transmission of Brownian motion* - facilitated by the relative (unidirectional) angular fluctuations of vanes immersed in different thermal baths and connected by the axle. Note that this represents a novel mode of heat transfer, distinct from the well-known mechanisms such as conduction, convection, and radiation.

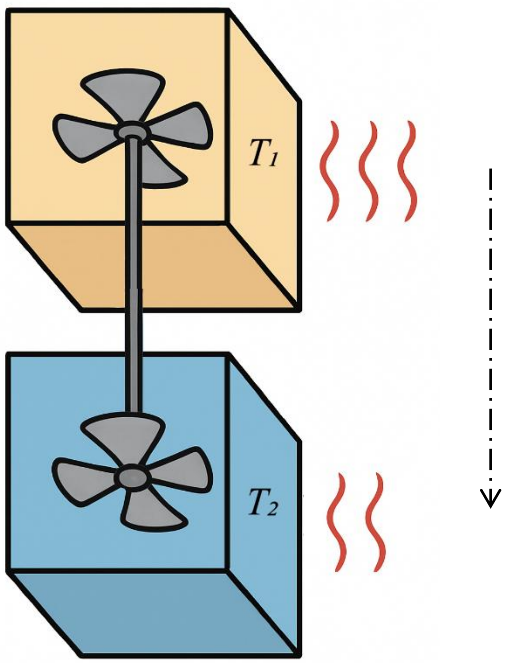

*Figure 1: The axle-vane engine, whose thermal conductance was originally formulated by Parrondo and Español [58] as a novel mode of heat transfer, and later used by us to estimate the thermal conductance of ATP synthase [7]. The engine consists of two vanes embedded in two thermally isolated boxes maintained at different temperatures ($T_1 > T_2$), connected by an insulated axle.*

The assumption of a thermally isolated axle does not suggest that analogous proteins in the IMM must be thermally insulated. Instead, it illustrates that even in this limiting case, heat transfer can still occur. In this section, we present the analogy between the axle-vane engine and ATP synthase, as introduced in our previous work [7]. In the following section, we extend this analogy to all other proteins embedded in the IMM. ATP synthase is the enzyme responsible for producing the majority of adenosine 5′-triphosphate (ATP) molecules in most living organisms during aerobic respiration. It is located in the inner mitochondrial membrane, the thylakoid membrane of chloroplasts, and the plasma membrane of bacteria. The enzyme consists of two main components: the Fo unit, which is embedded in the IMM, and the $F_1$ unit, which resides in the mitochondrial matrix (Figure 2

– left). Within the Fo unit, several subunits are involved, with the c-subunit and a-subunit playing key roles in capturing protons from the IMS and facilitating their translocation into the matrix (Figure 2 – left).

The significant pH difference across the IMM - acidic (high proton concentration) in the IMS and alkaline (low proton concentration) in the matrix - drives the binding of protons to various amino acid residues of the Fo unit, promoting forward proton translocation. Thus, the enzyme exhibits at least two forms of asymmetry: first, the proton concentration gradient, and second, the ratchet-like geometric asymmetry of the Fo and $F_1$ units. This unidirectional proton flow, combined with the unidirectional rotational motion of the $F_o$ unit (clockwise when viewed from the gap toward the matrix [59]), forms the basis of the analogy between the $F_o$ unit and a ratchet engine. This analogy has also been adopted by other researchers [48–52]. There is a trade-off between the rotational speed and efficiency of this enzyme when modeled as a ratchet engine - a relationship that has been analyzed by Wagoner and Dill from an evolutionary perspective [60].

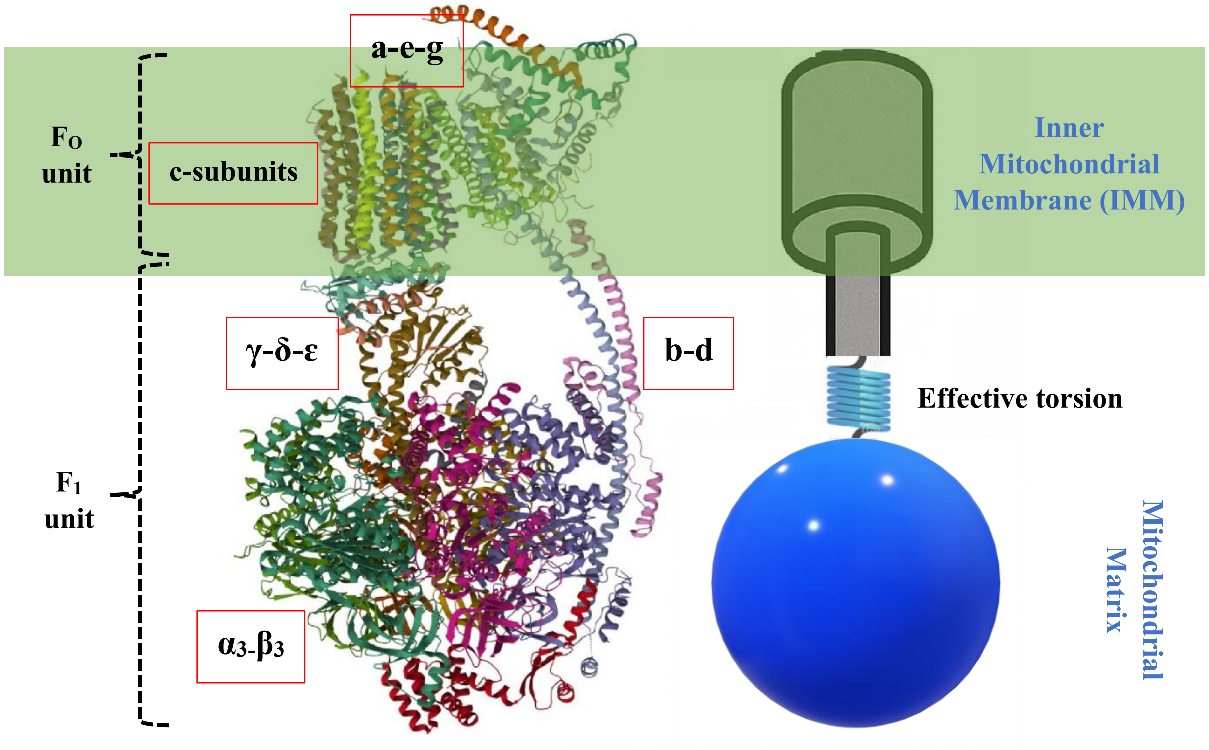

*Figure 2: The structure of human ATP synthase [61,62] containing eight c-subunits is shown in the left panel, with the Fo unit embedded in the inner mitochondrial membrane. The names of other major subunits are also indicated. A cartoon sketch on the right illustrates the modeled ATP synthase as a ratchet engine [7], where the γ-subunit is connected to the $\alpha_3\beta_3$-subunit via an effective torsional spring. The size of the c-ring in ATP synthases varies widely across species, with experimental evidence showing between 8 and 17 subunits [63]. A recent study used an AlphaFold-based computational approach to predict the stoichiometry of homooligomeric c-rings from genomic data, suggesting that naturally occurring c-rings could contain up to 27 subunits - far exceeding the experimentally observed range [64]. However, in our model, the number of c-subunits has minimal impact on temperature gradients. This is because the effective spring illustrated here is torsionally soft, and even a three-fold increase in the moment of inertia of the c-ring connected to the γ-subunit has a negligible effect on temperature. For detailed estimations, see page 104 of our previous work [7].*

Stochastic differential equations are widely used to model systems subject to random fluctuations, such as Brownian motion. In this framework, the deterministic equation of motion - typically Newton's second law stating that force equals mass times acceleration - is modified by adding random noise terms to account for fluctuations. This results in the Langevin equation, which incorporates both deterministic dynamics and stochastic influences. When the ratchet-engine model of ATP synthase is represented using the Langevin equation, the random collisions of protons in the IMS with the $F_O$ unit's amino acid side chains serve as the noise generator or "stochastic forces" [7]. The electrostatic torque responsible for rotation is generated when a proton enters the channel of the $F_o$ unit [65].

The ratchet-engine mode of heat transfer in ATP synthase results in very low thermal conductance due to friction between the enzyme and its environment - primarily arising in the γ-subunit within the $F_1$ unit (Figure 2, left) - as well as due to the enzyme's torsional properties. In this model, the torsional resistance is represented as an effective spring connecting the γ-subunit to the $\alpha_3\beta_3$ subunits (Figure 2, right), reflecting the torque required to rotate the enzyme by a given angle [7,66,67].

So far, we have discussed the thermal conductance of ATP synthase. We now turn our attention to the rate of heat production associated with proton translocation through the enzyme. Given that protons do not exist as such in aqueous media but that they rather exist as hydrated hydronium ions, and given that molecular-dynamic simulations suggest that only protons (not hydronium ions) are passed within ATP synthase's $F_o$ unit [68], for proton translocation to occur, the following process must be occurring (see also Figure 3). First, *endergonic processes occur on the IMS* since the hydration shell must first be stripped away from the hydronium ion followed by deprotonation of the hydronium ion to water and a proton. The proton is then captured by the amino-acid residues of the a-subunit or the a–c interface (the interface between the a-subunit and the c-subunit, Figure 3) of the $F_O$ unit, passing from one residue to the next within the channel until it exits on the matrix side of the IMM. The ejected proton in the mitochondrial matrix protonates a water molecule to form a hydronium ion, which is then solvated by the surrounding water, releasing the same amount of energy that was consumed on the other side of the membrane. The short-lived consumption of Gibbs energy in the IMS (mainly enthalpic under physiological conditions and thus referred to as 'heat') is followed by the release of an equivalent amount of heat in the mitochondrial matrix. Absorption of heat on one side of the membrane by an entering proton accompanied by the release of heat on the other side of the membrane by the exiting proton is presumed to result into an ultra-short spike of the temperature difference. (Note that the entry of a proton is simultaneous with the exit of the $n^{th}$ proton that entered the channel where $n$ is the number of c-subunits). This continual flow of protons through the channel where the entry of a proton is accompanied by the ejection of an "older" proton creates a decrease in the temperature at the IMS and simultaneously rise in the temperature at the matrix side.

Endergonic reactions at proton channel entry:

$H_3O^+(aq) + A(aq)$
$\rightarrow H_3O^+(g) + A(aq) + n(H_2O(aq))$
$\rightarrow H^+(g) + H_2O(g) + A(aq) + n(H_2O(aq))$
$\rightarrow H_2O(aq) + AH^+(aq)$

Exergonic reactions at proton channel exit:

$H_2O(aq) + AH^+(aq)$
$\rightarrow H^+(g) + H_2O(g) + A(aq) + n(H_2O(aq))$
$\rightarrow H_3O^+(g) + A(aq) + n(H_2O(aq))$
$\rightarrow H_3O^+(aq) + A(aq)$

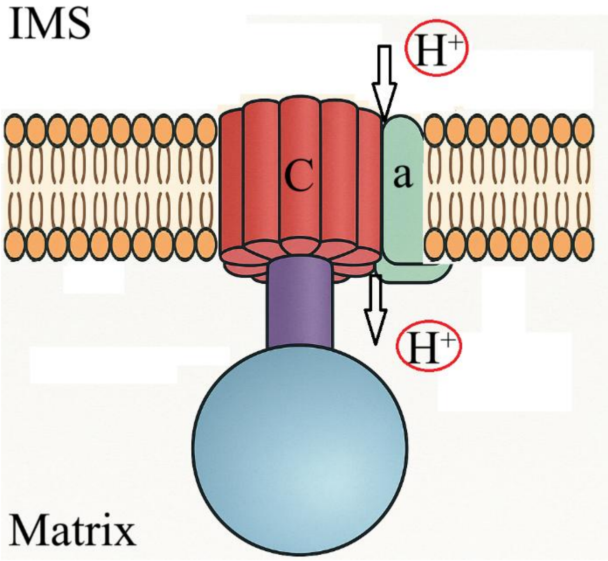

*Figure 3: The endergonic and exergonic reactions occurring at the entry and at the exit of the proton channel, respectively. (**Left**) $H_3O^+(aq)$ represents the hydronium ion in the aqueous phase, A(aq) denotes the first amino acid residue that captures the proton, (g) refers to the gas phase, $n(H_2O(aq))$ indicates the number of water molecules released during dehydration, and $AH^+(aq)$ is the final product of the reaction, showing that the proton has been successfully captured by the amino acid. (**Right**) A representation of this process, where protons enter through the interface between the c-subunit and a-subunit in the intermembrane space (IMS) and are subsequently released into the mitochondrial matrix after translocation.*

As explained in Ref. [7], estimating the temperature difference across the IMM requires a knowledge of the protonation energy of water ($\Delta G^0_{\text{Protonation}}$), the hydration energy of the hydronium ion ($\Delta G^0_{\text{Hydration}}$), the rate of proton translocation through ATP synthase ($N_{\text{p.u.t.}}$, for number of proton translocated per unit time), the torsional properties of the axel of ATP synthase ($\tau_{\text{eff.}}$), and the friction between the enzyme subunits and their local environments during conformational changes ($\lambda$), as shown in the following equation developed in [7] (the derivation of the equations used in [7] are provided in Chapter 1 of Ref. [10]):

$$\Delta T \cong \frac{2N_{\text{p.u.t.}}\lambda}{k_B\tau_{\text{eff.}}}\left(\Delta G^0_{\text{Protonation}} + \Delta G^0_{\text{Hydration}} + \Delta G^0_{\text{Heat bath}}\right) \qquad (1),$$

where $k_B$= 1.380649 × $10^{-23}$ J/K is the Boltzmann constant, and $\Delta G^0_{\text{Heat bath}}$ is the kinetic energy that the proton acquires from the thermal background of its surroundings upon translocation to the opposite side of the IMM. Upon binding to a water molecule, the proton transfers this kinetic energy to that molecule and its hydrogen-bonded neighbors through collisions. However, this energy is negligible compared to the free energy changes associated with protonation and hydration. *Equation (1) resembles Fourier's law but is formulated for a ratchet engine model, with parameter values specific to ATP synthase.* The term $\frac{2\lambda}{k_B\tau_{\text{eff.}}}$

corresponds to the inverse of thermal conductance, while $N_{\text{p.u.t.}}(\Delta G^0_{\text{Protonation}} + \Delta G^0_{\text{Hydration}} + \Delta G^0_{\text{Heat bath}})$ denotes the heat power. A detailed derivation is presented in Chapter 1 of [10] and in [7].

Note that we sum over all Gibbs free energy changes in Equation (1) because they occur within a few picoseconds - faster than the timescale of heat diffusion - which justifies treating them collectively. In other words, after analysis of the timescales of the various processes (protonation, and hydration) and the thermalization, the heat imbalance is estimated to thermalize within a few picoseconds [7].

The combined energy of water protonation and subsequent hydronium ion hydration amounts to approximately 1,200 kJ/mol (Table 1). The rate of proton translocation is $N_{\text{p.u.t.}} \approx$ 1,200 $H^+$/s, the friction coefficient of ATP synthase rotatory mechanism $\lambda \approx 4 \times 10^{-27}$ J·s/rad² [7], and the effective torsional constant of the axel is $\tau_{\text{eff.}} \approx$ 200 pN·nm/rad² [67]. The unit pN·nm/rad² stands for picoNewton–nanometer per radian squared, where force (pN) times distance (nm) is divided by the square of angular displacement (rad²). By inserting these parameters into Equation (1) [7], a short-lived temperature difference of approximately 6°C–7°C is estimated [7]. Note that the value of 1,200 $H^+$/s is based on a typical rotation speed of 150 revolutions per second [69] for ATP synthase with 8 c-subunits (150 × 8 = 1,200). However, rotation speeds as high as 350 revolutions per second have been reported at 37°C for *E. coli* ATP synthase, which has 10 c-subunits [70,71]. This could potentially result in a proton flux of 350 × 10 = 3,500 $H^+$/s, leading to a temperature difference of approximately 18°C–19°C across the thickness of the inner mitochondrial membrane, where the rate of heat production (which drives the temperature difference according to our theory (Eqn. (1) above or Eqn. (6) of Ref. [7])) is proportional to the rate of proton translocation.

## 3. Can IMM proteins other than ATP synthase be modeled as a ratchet engine?

In this section, we explore whether proteins of the inner mitochondrial membrane (IMM), aside from ATP synthase, can also be modeled as ratchet engines. Proteins within the IMM - such as ion exchangers, mitochondrial carrier proteins (MCPs), uncoupling proteins (UCPs), ABC transporters, and respiratory complexes (for a comprehensive review of ion channels in the mitochondrial membrane, see references [72,73]) - all share a common mechanism of energy conversion and transport that is consistent with the behavior of ratchet engines. These proteins use gradients of ions and metabolites to facilitate unidirectional transport, driven by random collisions and conformational changes under non-equilibrium conditions. The modeling of ion pumps (such as $Na^+/K^+$-ATPase [55] and the bacteriorhodopsin proton pump [53,54]) and voltage-dependent ion channels [56] as ratchet engines has been extensively explored in the literature.

In physical models of ratchet engines [49,57] and axle-vane engines [58], the random Brownian motion of particles is assumed to directly drive engine rotation - for example, the rotation of the c-subunits in ATP synthase as described in the previous section. Here, we extend the definition of physical ratchet engines to include other IMM proteins. Ions undergoing Brownian motion frequently collide with these proteins. When an ion is recognized and captured by a protein, the protein can harness this interaction to perform work, often manifested as "*conformational changes*". These conformational changes, in turn, drive the protein's functional or mechanical activity facilitating the translocation of the ion.

Consider the three-dimensional IMM as being composed of an infinite number of segments. Each segment maintains an ion gradient (e.g., $H^+$, $Na^+$, $Ca^{2+}$) across the membrane, either from the matrix to the IMS or *vice versa*. Proton gradients, for instance, drive proton pumps in respiratory complexes through the electron transport chain (ETC) to move protons from the matrix to the IMS. Simultaneously, other ions, such as $Na^+$ and $Ca^{2+}$, are exchanged as required by physiological conditions. Proteins of the IMM function unidirectionally *for a given ion gradient and a given time* consistent with the ratchet-engine model. One might question whether ion exchangers, such as the sodium-proton exchanger in an antiport system, exchange ions simultaneously, which would seem to contradict unidirectionality. However, even in this type of channel, the exchange does not occur exactly at the same time, as the protein cannot undergo two different conformational changes simultaneously. Instead, the processes occur in succession, *making each individual event unidirectional in time*.

The conformational changes that facilitate ion transfer are influenced by factors such as the friction between the moving protein subunits and their surrounding environment, as well as the torsional stiffness of the protein. Protein torsion depends on the mechanical properties of the protein, particularly the stiffness of the subunit undergoing conformational changes that facilitate ion translocation. Additionally, the friction between the protein and its membrane-bound environment varies based on hydrodynamic, hydrophobic, and steric interactions, which can differ from one protein to another. It is also important to note that the micro-viscosity of the IMM is not constant; it varies with the electric field strength of the membrane, which is influenced by the cell's energy status - whether satiated or starved [74]. Thus, an actively growing cell with a higher proton motive force across the IMM generates a stronger electric field, which in turn increases the IMM's viscosity and friction [74]. This can potentially lead to a greater temperature difference across the two ends of proteins within the IMM during ion translocation, as temperature difference correlates positively with friction, according to Equation (1).

When the concentration gradient of a specific ion is higher on one side, the collision frequency of those ions with the corresponding protein increases. In terms of the ratchet model, *an ion gradient is analogous to a temperature gradient*, as it drives directional work through stochastic processes. For ions to be translocated across membranes via channels and pumps, they are generally (at least partially) dehydrated in order to be "*felt*" by the channel [75]. This is well-documented in the cases of sodium and potassium channels [76,77]. An exception occurs when the pore is sufficiently wide to allow ions to pass through with their hydrated shells intact [78]. Once an ion is dehydrated, it is captured by amino-acid residues within the protein channel through random Brownian collisions. These interactions induce conformational changes in the protein, facilitating the ion's translocation to the other side of the membrane, where it rehydrates with water molecules.

The energy of hydration varies for different ions and metabolites, depending on their charge, size, and chemical properties and is crucial in determining the temperature difference across the IMM (see Eqn. (1) above and details in Ref. [7]). Given the role of hydration (and dehydration) in the transport of ions through biological membranes, in Table 1 we list known approximate Gibbs energies of hydrations at standard temperature (298.15 K) and pressure (1 atm) for common ions and metabolites encountered in cellular processes. Unlike the protonation energy, which is a well-defined chemical reaction where one hydrogen ion ($H^+$) combines with a water molecule ($H_2O$) to form a hydronium ion ($H_3O^+$) in a one-to-one ratio ($H_2O + H^+ \rightarrow H_3O^+$), hydration doesn't follow a fixed formula. This is because hydration refers to how water molecules surround and interact with a solute (like an ion or molecule) in solution. These water molecules form layers (called solvation shells) around the solute, but the exact number and arrangement of water molecules are not fixed. They change constantly due to the motion and interactions in the liquid, making hydration a statistical and dynamic process as captured for instance in molecular-dynamics simulations where it is typical to plot distribution functions of solvent molecules around the solute molecule or ion [79,80].

Table 1: Gibbs energies of hydration of some common ions in the cellular medium.

| Ion | Δ*G*(hydration), kJ/mol | Ref. |
|---|---|---|
| $H^+$ | ~ -1183[(a)] | [81] |
| $Na^+$ | -406 | [82] |
| $K^+$ | -322 | |
| $Ca^{2+}$ | -1577 | |
| $Mg^{2+}$ | -1921 | |
| $Cl^-$ | -363 | |
| $HCO_3^-$ | -335 | [83] |
| $PO_4^{3-}$ | -2765 | |

(a) This value is for the sum of Gibbs energies of protonation and of hydration.

If ion translocations by one protein are staggered with those of neighboring ones, temperature difference across the IMM would be continuous over time in this neighborhood. There are no apparent reasons to rule-in or rule-out the synchronization of ion translocation undertaken by two or more independent protein molecules. However, cristae membranes are tightly packed, densely populated with proteins, creating *confined spaces* that may retain heat locally for extended periods. An ensemble of such proteins would have translocation events at a rate $P \approx \sum_{i=1}^{n} N_i M_i s^{-1}$ where $N$ is the number of ions translocated per second per protein molecule, $M$ is the copy number of such protein molecules in the neighborhood sensed by the fluorescent probe, and $n$ is the number of states.

Higher temperature differences could be achieved if the rate of ion translocation and/or the hydration energy for other ions are higher than those for protons in ATP synthase. In other words, any membrane - whether of an organelle or a cell - that is highly selective and contains a high protein-to-lipid ratio can potentially become "hot", provided the interplay of ion translocation rates, hydration events, protein torsion, and membrane friction generates a significant temperature gradient.

The rate of ion translocation in ion channels, such as potassium channels, is ~ $10^8$ ions/s [84], five orders of magnitude higher than the rate of proton translocation through ATP synthase. This raises the question of whether it results in a temperature difference five orders of magnitude greater than the one we estimated for ATP synthase. The answer is no, primarily because the rate of translocation depends on the friction between the enzyme and its surrounding membrane lipids. The torsional friction for a full rotation of ATP synthase is as high as 40 pN·nm [85], which significantly impedes the rotation of the enzyme [71]. The friction opposing conformational changes in an ion channel must be significantly lower than that involved in the full rotation of ATP synthase, allowing the ion channel to support a much higher rate of ion translocation. Thus, the high rate of transport by ion channels is cancelled out by the extremely low friction against their conformational changes (see Eqn. (1)), resulting in temperatures similar to those we estimated for ATP synthase. Although we currently lack direct measurements of the torsional stiffness of ion channels and the friction opposing their conformational changes, the theoretical hydrodynamic model developed by Chen *et al.* [86], which is distinct from ours, predicts that the translocation of a single sodium ion through the gramicidin A channel (25 Å in length and 2 Å in radius) under a 100 mV transmembrane potential could result in a local temperature increase of 25 °C per ion.

## 4. Can computational modeling shed light on time & length scale of temperature responsiveness of fluorescent probes?

Experimental fluorescence thermometry is limited to millisecond-scale resolution [87,88] which reflects instruments constraints and not necessarily the intrinsic response time of fluorescent probes. It remains an open question whether these molecular thermometers can sense rapid, localized temperature spikes on picosecond to nanosecond scales as those caused by ion translocation events in mitochondria.

On one hand, quantum chemical calculations offer a path to investigate this possibility. *Ab initio* (and post-Hartree-Fock) calculations [89–92], density functional theory (DFT) [93–95], and time-dependent DFT (TD-DFT) [93–95] can model the photophysical properties of fluorescent dyes' response to sudden thermal perturbations. TD-DFT, for example, can predict how excitation energies and oscillator strengths shift due to temperature-induced structural changes occurring within the nanosecond timescale of the dye's excited-state lifetime. On another hand, quantum molecular dynamics (e.g., Car-Parrinello MD simulations [96,97]) can probe the spatial range of thermal effects, revealing how hydration shells reorganize around the dye in response to localized heating. While the experimental signal is presumed to reflect an integral of the signals from many probes over time and space, the underlying emission could still reflect these nanoscale thermal spikes.

## Closing Remarks

This article revisits the "*Hot-Mitochondrion Paradox*" (HMP), the apparent contradiction between experimental evidence suggesting mitochondria operate ~10-15°C hotter than their environment and theoretical limits imposed by classical steady-state heat transfer as stipulated by Fourier's law. The fluorescent thermometry results of Chrétien *et al.* suggest a six-order-of-magnitude discrepancy from steady-state Fourier-law prediction of the temperature difference across the IMM, known in the literature as the "$10^5$ gap". We propose resolving this paradox by modeling the proteins of the IMM as Brownian ratchet engines that generate ultrashort, localized bursts of heat during proton or ion translocation events. Our model predicts short-lived (~picosecond) temperature-difference spikes on the order of 6°C which can even potentially reach ~ 20°C, consistent with experimental fluorescence thermometry. We further suggest that the cumulative action of numerous membrane proteins with ion transport capability can create persistent localized temperature differences in a given neighborhood of a fluorescent dye molecule. The framework developed in [7] is generalizable to other membranes with a high protein-to-lipid ratio and implies broader implications for thermally mediated cellular processes. The ratchet model coupled with the dehydration-translocation-rehydration sequence of events and the presence of a large number of proteins thus offers a physically plausible and mechanistically sound reconciliation of theory with observation. If mitochondrial membrane proteins generate short-lived temperature spikes, is this something evolution has adapted for (to speed up reactions) or is it merely an unavoidable byproduct of biological processes? It's likely an inevitable byproduct, but evolution may have taken advantage of it where useful. Think of it like a car engine: the engine gets hot as a side effect of running. Engineers might place heat-sensitive components near the engine to make use of that heat. But the heat isn't the goal, it's a byproduct that can sometimes be useful.

It is fitting to conclude with a quote from Sainsbury's 1990 book on Paradoxes:

> "This is what I understand by a paradox: an apparently unacceptable conclusion derived by apparently acceptable reasoning from apparently acceptable premises. Appearances have to deceive, since the acceptable cannot lead by acceptable steps to the unacceptable. So, generally, we have a choice: either the conclusion is not really unacceptable, or else the starting point, or the reasoning, has some non-obvious flaw.
>
> Paradoxes come in degrees, depending on how well appearance camouflages reality. Let us pretend that we can represent how paradoxical something is on a ten-point scale. The weak or shallow end we shall label 1; the cataclysmic end, home of paradoxes that send seismic shudders through a wide region of thought, we shall label 10".

According to this scale, we imagine that the hot mitochondrion paradox, where theory and experiment disagree neither by a factor of 2 factor nor by a factor of 100, but rather by a factor of one million deserves a place at the summit of the pyramid of paradoxes with a well-deserved label of 10.

## Acknowledgments

The authors thank Professor Thanh-Tung Nguyen-Dang (Laval University) for his helpful discussions. CM and PF are grateful to the Natural Sciences and Engineering Research Council of Canada (NSERC), the Canada Foundation for Innovation (CFI), Saint Mary's University, Dalhousie University, Mount Saint Vincent University, and the Digital Research Alliance of Canada for their financial support and resources. ML is grateful to the National Institutes of Health (2R35GM122566), the National Science Foundation (DBI-2119963, DEB-1927159), the Simons Foundation (735927), and the Moore Foundation (12186).
